# Diffusion-Driven Charge Transport in Light Emitting Devices

**DOI:** 10.3390/ma10121421

**Published:** 2017-12-12

**Authors:** Iurii Kim, Pyry Kivisaari, Jani Oksanen, Sami Suihkonen

**Affiliations:** 1 Department of Electronics and Nanoengineering, Aalto University, P.O. Box 13500, 00076 Aalto, Finland; sami.suihkonen@aalto.fi; 2 Engineered Nanosystems Group, Aalto University, P.O. Box 12200, 00076 Aalto, Finland; pyry.kivisaari@aalto.fi (P.K.); jani.oksanen@aalto.fi (J.O.)

**Keywords:** light-emitting diodes (LEDs), diffusion injection, lateral epitaxial overgrowth, selective-area growth (SAG)

## Abstract

Almost all modern inorganic light-emitting diode (LED) designs are based on double heterojunctions (DHJs) whose structure and current injection principle have remained essentially unchanged for decades. Although highly efficient devices based on the DHJ design have been developed and commercialized for energy-efficient general lighting, the conventional DHJ design requires burying the active region (AR) inside a pn-junction. This has hindered the development of emitters utilizing nanostructured ARs located close to device surfaces such as nanowires or surface quantum wells. Modern DHJ III-N LEDs also exhibit resistive losses that arise from the DHJ device geometry. The recently introduced diffusion-driven charge transport (DDCT) emitter design offers a novel way to transport charge carriers to unconventionally placed ARs. In a DDCT device, the AR is located apart from the pn-junction and the charge carriers are injected into the AR by bipolar diffusion. This device design allows the integration of surface ARs to semiconductor LEDs and offers a promising method to reduce resistive losses in high power devices. In this work, we present a review of the recent progress in gallium nitride (GaN) based DDCT devices, and an outlook of potential DDCT has for opto- and microelectronics.

## 1. Introduction

The electrically driven double heterojunction (DHJ) sandwiching an active material layer between the p- and n-type charge injection layers is nowadays so ubiquitous in semiconductor industry that it is almost impossible to imagine any viable options for it [1,2]. Particularly, all laser diodes and highly effective light-emitting diodes (LEDs) [3], as well as many heterostructure bipolar transistors [4], field effect transistors [5], and state-of-the-art solar cells [6] use DHJs whose function has remained essentially similar for decades. All of these structures traditionally realize the current transport by using the conventional DHJ-like configuration, where the active region, e.g., quantum well (QW) or multi-quantum well (MQW) stack, is located between n- and p-doped semiconductor regions and the electrons and holes enter the active region from the opposite directions. In the case of LEDs, biasing the LED generates a drift current transporting carriers into the opposite edges of the depletion region. The charge carriers are further transported and spread in the active region by diffusion. This configuration satisfies the needs of most LED structures for general lighting. Nevertheless, conventional LEDs still come across with some technological and power efficiency challenges, especially in high-power lighting applications [2,7].

Sandwiching the active region (AR) between the n- and p-type regions is straightforward with modern fabrication processes, but imposes limits on the device geometries that can be realized without effort [8,9]. Moreover, utilizing modern materials such as nanowires (NW), quantum-dots (QDs), surface plasmon enhanced and 2D-materials for the active region is both interesting for research and promising to enhance LED performance. However, the complications arising from the LED design based on the DHJ model are a significant bottleneck for utilizing such new materials [10,11,12]. For example, around 40 years passed since the invention of the nanowire growth mechanism [13] before the first NW based LED was fabricated [14,15,16]. If DHJ is used, the NW must have contacts on both ends to enable an electrical path through the nanowire, and thus contact fabrication for NWs becomes challenging due to the long and complicated process. In addition, the deposition of top contact, in general, absorbs the light emitted by any materials listed above decreasing the efficiency of the LED.

From the III-nitride LED point of view in particular, the phenomenon called ”efficiency droop” is assessed as one of the most prominent scientific and technological challenges [17]. In the droop-effect, an increasing injection current leads to significant drop off in the emission efficiency of blue LEDs with indium gallium nitride (InGaN) MQW active layers [18,19,20]. The mechanisms of the efficiency droop in InGaN LEDs have been studied extensively, where carrier delocalization [21,22,23] and electron leakage [18,24] are proposed to be key reasons, while the most recent reports mainly pointing to Auger recombination as the main culprit [25,26,27,28]. Secondly, particularly in the modern high quantum efficiency LEDs, the efficiency droop limitations, current crowding and resistive loss become the most severe bottlenecks for high output power devices, confining their optimal high-efficiency performance at current densities well below 100 A/cm${}^{2}$ [29,30,31,32,33,34].

Diffusion-driven charge transport (DDCT) has been recently developed as a possible alternative current injection method in order to avoid DHJ limitations [35,36], originally with the aim to enable efficient current spreading over large area light emitters for electroluminescent cooling devices such as thermophotonic heat pumps [37]. In contrast with conventional DHJ, in the DDCT-scheme, the AR is located outside the pn-junction, and both carrier types (electrons and holes) diffuse to the AR through at least partly overlapping paths. Following the originally computational introduction of the DDCT-scheme, the III-nitride diffusion injected light emitting diode (DILED) [38,39,40] and surface InGaN QW located on top of gallium nitride (GaN) pn-homojunction (S-LED) [41] have been fabricated and characterized. It has been demonstrated that the obtained devices lean on the mechanism of carrier diffusion to the QW/MQW excited through one of its interfaces only. In addition, simulations suggest that the efficiency of DDCT devices based on lateral heterojunctions (LHJ) [42] can also exceed the efficiency of comparable DHJ structures. Consequently, the DDCT-scheme can offer new possibilities for high-power lighting applications as well as several emerging devices making use of nanowires, 2D materials, quantum dots, plasmonic and near field phenomena. In this study, we review the recent progress in light-emitting diodes based on diffusion-driven charge transport. We also discuss the outlook for using DDCT to electrically excite new promising materials such as monolayers and quantum dots.

## 2. First Demonstrations of Diffusion-Driven Charge Transport

### 2.1. Basics of the DDCT Concept

In principle, conventional electrical excitation of LEDs as well as the structures mentioned above are based on an AR sandwiched in a pn-junction. When the LED is biased, the transport of majority carriers mainly takes place as a drift current due to a small electric field transporting the carriers from the n- and p-type regions towards the depletion region. Starting from the edge of the depletion region, the main component of the net current, on the other hand, is diffusion. Therefore, typical LEDs essentially behave as 1D structures where diffusion transports electrons and holes to the AR from p- and n-type regions located at opposite sides of the AR. However, the minority carrier diffusion may extend over relatively long distances even in the presence of the DHJ potential barriers. This represents a main disadvantage for conventional devices, so far as diffusion of electrons over the MQW results in carrier leakage and decreases the device efficiency. In contrast, DDCT-based devices take advantage of such diffusion currents.

**Figure 1:**
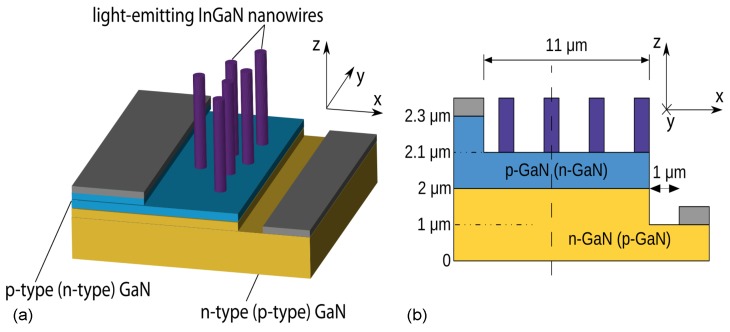
(**a**) schematic illustration of the free-standing n-type (p-type) nanowire emitter structures studied in Ref. [35]; (**b**) the two-dimensional lateral cross section model of the structure and dimensions as they are used in the calculations of the reference. Note that the figures are not in scale. The nanowires are placed on top of the pn-junction and injected by bipolar diffusion. Reproduced from [35], with the permission of ©AIP Publishing 2013.

Diffusion-Driven Charge Transport was originally introduced in Refs. [35,36] as a current injection scheme for nanostructures, where the active region was located outside the pn-junction and the conventional current path. DDCT is based on (1) utilizing the strong diffusion currents predicted by the Shockley diode equations and (2) having a smaller bandgap AR that acts as a sink for the diffusing carriers so that combining these two allows electrical excitation of ARs located outside the pn-junction. In Ref. [35], we presented numerical solutions of current transport equations for freestanding nanowire emitter structures based on III-N semiconductors shown in Figure 1. It was suggested that bipolar diffusion injection works with both minority electrons and minority holes, by a comparing two different variations where the thin bulk region immediately below the nanowires was either p- or n-type.

The ultimate requirement to make use of DDCT is that the AR is located within the diffusion length of carriers (electrons or holes) from the pn-junction. This will enable a diffusion path for minority carriers between the pn-junction and the AR. As an example, in Ref. [35], the structure simulated under a 3.5 V bias resulted in substantial electron/hole densities in the NWs. Specifically in the structure with p-GaN below the NWs, the electron concentration was small in the p-type region and large in the NWs, resulting from efficient diffusion of electrons through the p-type region from the pn-junction. Similar effect was obtained for the reverse structure where diffusion injection worked with minority holes. Moreover, it was shown that due to large electron and hole concentrations in the NWs, almost all recombination took place there. These results suggested that the bipolar diffusion injection concept can be used to inject free-standing nanowire structures, and they encouraged us to test the idea experimentally, first with planar GaN LEDs with InGaN QWs.

### 2.2. Theory and Equivalent Circuit

**Figure 2:**
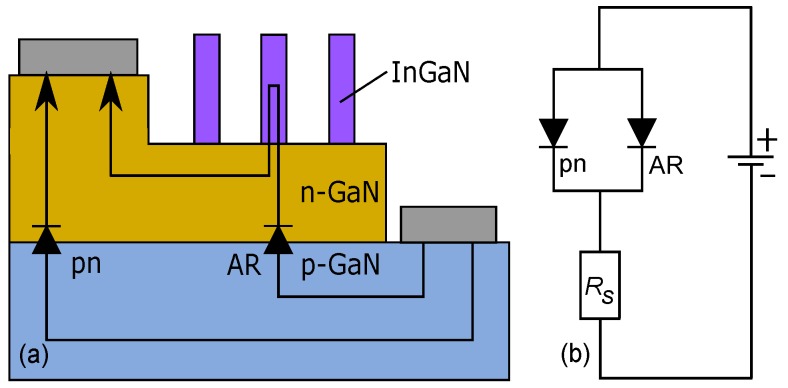
(**a**) simplified sketch of one of the structures studied in Ref. [35] and (**b**) its equivalent circuit model. The equivalent circuit has two parallel diodes describing leakage current to the contacts (labelled “pn”) and current to the active region. The resistance $R_{s}$ describes resistive losses in the homogeneous regions of the device.

The basic features of DDCT can be explained using standard semiconductor transport models summarized e.g., in Ref. [42]. For illustration purposes, in Ref. [39], we also developed an equivalent circuit model to study how the diffusion current to the AR and the loss currents depend on the structure details, and how the current to the AR can be enhanced. Here, we summarize the model for the structure shown in Figure 2a with its equivalent circuit shown in Figure 2b. The device consists essentially of the pn-junction in the GaN host material and the lower-bandgap InGaN AR, both of which can be modelled as parallel diodes with their separate diode laws. If the host material has a relatively low number of defects, the pn-junction current consists primarily of carrier diffusion to the contacts, which can be approximated with the short diode law given by

(1) $$I_{pn}=qn_{i}^{2}\left(\frac{D_{n}A_{p}}{N_{a}L_{p}}+\frac{D_{p}A_{n}}{N_{d}L_{n}}\right)\left[\exp\left(\frac{qV_{pn}}{k_{B}T}\right)-1\right],$$

where *q* is the elementary charge, $n_{i}$ is the intrinsic carrier concentration, $D_{n,p}$ are the diffusion constants of electrons and holes, $A_{n,p}$ are the cross-section areas of the n- and p-contact, $N_{d,a}$ are the ionized donor and acceptor densities, $L_{n,p}$ are the distances between the pn-junction edge and the n- and p-type contacts, $V_{pn}$ is the voltage applied over the pn-junction, $k_{B}$ is Boltzmann’s constant, and *T* is the temperature.

When the device includes a low-bandgap AR outside the pn-junction as in Figure 2, recombination in the AR forms a somewhat similar current sink as the carrier loss taking place at the two contacts which resulted in Equation (1). In the device of Figure 2, there is always a large density of electrons next to the AR, and recombination in the AR is therefore limited by the availability of holes. In this case, holes can diffuse from the pn-junction to the AR similarly as when they diffuse towards the n-contact, and this hole diffusion can be approximated with a diode law reminiscent of Equation (1), given by

(2) $$I_{AR}=qn_{i}^{2}\frac{D_{p}A_{AR}}{N_{d}L_{AR}}\left[\exp\left(\frac{qV_{pn}}{k_{B}T}\right)-1\right],$$

where $A_{AR}$ is the cross-section area of the AR and $L_{AR}$ is the distance between the pn-junction edge and the AR. Comparing Equations (1) and (2), it can be seen that $I_{AR}$ can be increased without increasing $I_{pn}$ e.g., by extending the cross-section area of the AR and decreasing the distance between the AR and the pn-junction edge. If most of the pn junction current consists of electron leakage as is usually the case with GaN, $I_{AR}$ can even be enhanced by decreasing $N_{d}$. On the other hand, increasing temperature is expected to enhance the operation of the structure in Figure 2 partly by increasing $D_{p}$ and, in the case of GaN, more importantly by enhancing acceptor activation and hence the number of holes available for diffusion. In the context of Equation (2), the increasing acceptor activation decreases $L_{AR}$, as the depletion region extends further to the n-side and its edge therefore moves closer to the AR. On the other hand, in Section 3, we analyze devices that can enhance $I_{AR}$ significantly further by using lateral doping techniques.

Please note that recombination taking place in the host material is not included in the loss current in Equation (1). However, recombination in the host material is orders of magnitude smaller than recombination in the AR due to the smaller bandgap and consequently much larger carrier densities in the AR. In other words, even if both electrons and holes are present in the pn-junction, the rate of carrier diffusion towards the AR is much faster than the rate of recombination in the pn-junction where the carrier densities are much lower than in the AR. If, however, the host material is of poor quality, the defect recombination may constitute another significant loss current mechanism similarly as in any DHJ-based device that has a poor material quality and a large number of defects. An interesting additional feature differentiating between the bipolar diffusion injection and conventional current injection is that due to the equal electron and hole fluxes to the AR, the current through any horizontal AR cross-section is zero.

### 2.3. Diffusion Injected Buried MQW LED (DILED)

**Figure 3:**
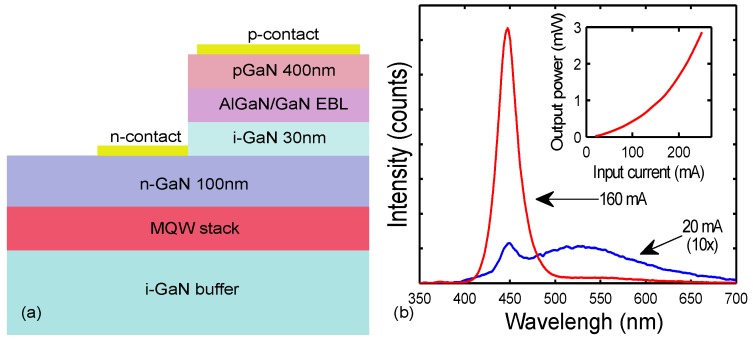
(**a**) schematic illustration of the layer structure and thicknesses of the diffusion injected buried the multi-quantum well (MQW) LED studied in [38]. This was the first experimental demonstration of carrier injection to the MQW AR by bipolar diffusion. The InGaN/GaN MQW stack is located under both p- and n-layers and thus outside the pn-junction; (**b**) spectra of the studied diffusion injected LED (DILED) at injection currents of 20 mA and 160 mA measured at room temperature showing the emission from the MQW at 450 nm. The intensity of the 20 mA measurement is scaled by a factor of 10 in order to show the lineshape of the spectrum. The measured optical power as a function of input current is shown in the inset. Reproduced from [38], with the permission of ©AIP Publishing 2014.

As the first experimental verification of the DDCT concept, in Ref. [38], we reported the first buried multi-quantum well light-emitting diode structure injected by the bipolar diffusion. The fabricated device contained a MQW stack located below the GaN pn-junction as schematically illustrated in Figure 3a. The device structure was based around the conventional III-nitride LED fabrication processes and utilized the same metal-organic chemical vapor deposition (MOCVD) growth, lithography, etching and contacting steps. The MQW stack was placed under the n- and p-doped regions in order to avoid the magnesium (Mg) memory effect [43,44,45] in MOCVD and to avoid a dry etching of the p-doped layer. The electrically excited sample showed a strong blue emission at room temperature (300 K) at 450 nm wavelength with 160 mA injection current, corresponding to the emission from the InGaN AR. With low excitation power (20 mA), also yellow luminescence was observed and identified to result from defects in the unintentionally doped GaN (i-GaN) spacer between the p- and n-GaN. However, since the QWs were located outside the pn-junction, blue emission confirmed that both electrons and holes were transported to the QWs from the same side of the active region through bipolar diffusion. Secondary electron-hole generation in the MQW due to UV light emission from the pn-junction was ruled out as there was no trace of band-edge luminescence from the pn-junction in the spectrum (Figure 3b), meaning that the excitation level in the pn-junction was still weak.

Figure 3b shows the measured optical output power of the sample as a function of the injection current. As can be seen from the figure, the output power of the DILED increased superlinearly with increasing input current. This exceptional behavior, i.e., no effect from efficiency droop at high injection currents was explained by a low carrier concentration in the active region, so that the LED did not yet enter the droop regime.

The electrical and optical properties of fabricated buried MQW DILED were studied more extensively in Ref. [39]. We demonstrated that, with increasing temperature, the emission intensity is also increased in contrast to conventional LEDs, where the intensity typically decreases. This was found to be related mainly with the activation energy of the p-type Mg acceptors, which is relatively high in Mg doped p-GaN and results in low acceptor activation at room temperature. Increasing device temperature increases the acceptor activation and thus the hole diffusion current through n-GaN. The hole diffusion current can be thought as the main factor limiting the device efficiency. Effects of Mg doping on the temperature characteristics of typical III-N LEDs have been investigated e.g., in Ref. [46]. In Ref. [40], a similar device structure was studied with the exception that the AR consisted of five InGaN QWs with varying indium composition. Electroluminescence from each InGaN QWs was observed indicating that bipolar diffusion can not only excite the QW nearest to a pn-junction, but also to transport both electrons and holes over the potential barriers of a MQW stack.

The presented structures were the first experimental demonstration that bipolar diffusion can transport electrons and holes into the active region located outside the pn-junction and a proof of the diffusion-driven charge transport concept. This device configuration was designed to demonstrate the basic operating principle of diffusion injection by modifying a conventional III-nitride LED fabrication processes. The efficiency of the device was fairly low, but simulations suggest that relatively simple methods can be used to significantly increase the efficiency of the DILED structure by modifying its geometry and doping levels, bringing the injection efficiency even close to unity [39]. However, the structure shown in Figure 3 did not completely exclude the possibility of an alternative current path, as electrons enter the intrinsic GaN below the MQW and enter the MQW from the bottom side below the p-contact.

### 2.4. Diffusion-Driven Surface QW LED (S-LED)

While the diffusion injected buried MQW LED described in the previous chapter was the first experimental demonstration of a DDCT-based LED, it had very little novel device functionality. The further development of DDCT-based devices had a double motivation. On the one hand, the goal was to eliminate all parallel non-diffusion based current paths, and, on the other hand, to demonstrate the novel possibilities enabled by the DDCT-structure. The current diffusion-driven charge transport model can be applied to solve design challenges related to emitters based on near surface quantum wells, surface NWs, QDs, and layered 2D emitting materials, which are hard-to-reach with conventional DHJ structures. With the help of DDCT, such emitters can be excited electrically through the bottom contact only with no need for top contacts. This will allow integration of nano-scale surface light emitters in applications which are impossible to realize with DHJ. Moreover, a device with a surface AR leaves out all other electrical excitation mechanisms except bipolar diffusion through only one side of the AR.

**Figure 4:**
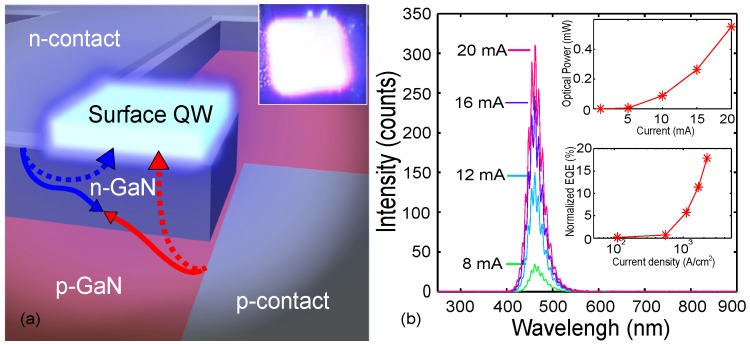
(**a**) the S-LED structure where a single quantum well (QW) is placed on the device surface and excited by bipolar diffusion [41]. The figure also illustrates the drift (solid line) and diffusion (dashed line) current components for electrons (blue) and holes (red). Microscope images of the S-LED under electrical excitation with injection current of 20 mA shown in the inset; (**b**) emission spectrum with insets of the optical output power at room temperature, and normalized external quantum efficiency with the reference double heterojunction (DHJ) device. Reproduced from [41], with the permission of ©AIP Publishing 2015.

To pursue these goals, our group recently demonstrated the diffusion injection excitation for near surface light-emitting structures [41]. The fabricated S-LED is illustrated in Figure 4a and contains an InGaN QW located on top of a GaN pn-junction. Such design is leaving the light-emitting surface entirely free of metals or other contact structures. The electrically excited charge carriers from the pn-homojunction are transported to the near surface QW by the bipolar diffusion as indicated by arrows in Figure 4a. In addition, the structure does not enable any alternative charge transport paths to the AR than bipolar diffusion from the same side of the AR.

As in the DILED structure of the previous subsection, the light emission from the S-LED is made possible by functionally separating the pn-junction which initially creates the excitation and the near surface QW where the radiative recombination takes place. In suitably engineered structures, the carriers injected into the p- and n-layers are efficiently transported to the near surface QW by bipolar diffusion through the bottom interface of the QW only. Therefore, there is no carrier flux through the top interface of the QW and the net current through any horizontal cross-section of the QW is always zero as in optical pumping. However, in contrast to direct optical pumping, the demonstrated electrical excitation method does not directly generate carriers in the surface quantum well, but all the carriers instead enter the QW through bipolar diffusion.

Conclusive proof of the diffusion current injection was observed in the emission spectrum of the electrically driven device (Figure 4b). As in the case of the buried QW device in the previous subsection, the intense light emission from the InGaN QW and the absence of any band-to-band UV emission from the GaN layers clearly showed that the charge carriers were transported to the QW through its bottom interface by diffusion. Moreover, strong blue emission was easily observed by a naked eye at room temperature (inset of Figure 4a). The external quantum efficiency (EQE) of S-LED is approximately one fifth of the efficiency of a reference single QW InGaN/GaN DHJ device at room temperature shown in the inset of Figure 4b. This corresponds to an optical power of 0.5 mW from the 30 × 30 μm QW mesa at 20 mA operating current as demonstrated on the second inset of Figure 4b.

The S-LED clearly shows that a surface QW can be excited by carrier diffusion through the bottom interface of the QW only. As the first demonstration of an electrically injected near surface QW, the S-LED provides the conclusive evidence of its feasibility for exciting surface nanostructures.

## 3. Laterally Doped DDCT Devices

All structures mentioned above as well as the associated theory and simulations were built on a vertically formed pn-homojunction. Presented devices demonstrated the first experimental verification of the DDCT concept and its potential to solve different design challenges in conventional LEDs. Nonetheless, the vertically formed pn-homojunction model involves potential barriers and leads to electrical inefficiencies that do not fully support reducing the effects of current crowding, resistive losses and efficiency drop.

### 3.1. Lateral Heterojunction (LHJ) Concept

**Figure 5:**
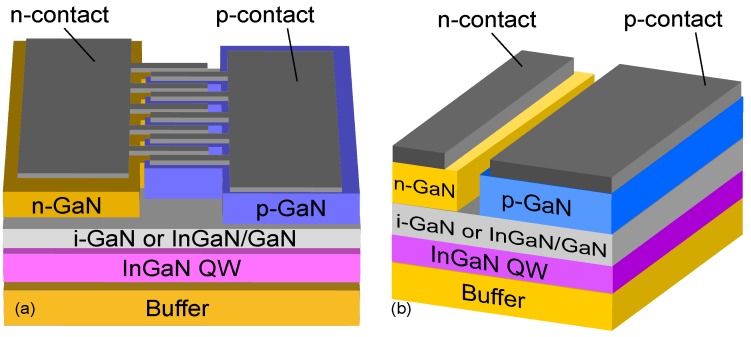
Schematic illustration of the lateral heterojunction (LHJ) LED finger structure, where the lateral pin-junction is used instead of a vertically formed pn-junction to improve carrier diffusion to the QW [42]. (**a**) perspective image of the full chip and (**b**) side view between two fingers. Figure 5b reproduced from [42], with the permission of ©Wiley-VCH Verlag GmbH & Co. KGaA. Publishing 2017. John Wiley & Sons, Ltd. Publishing 2017.

An exciting alternative to the vertical design could be offered by a structure with a lateral pin-junction such as the one shown in Figure 5. The structure consists of an active region with laterally overgrown GaN layers fabricated e.g., with selective area regrowth. We presented the first steps towards the realization of such planar design in Ref. [42,47]. Simulations suggested that electrical inefficiencies and sub-optimal device performance observed in previous studies can be eliminated by adapting the DDCT concept in laterally doped heterojunction (LHJ) structures. Figure 5 shows a schematic illustration of LHJ structure, where narrow n- and p-doped regions are fabricated side by side, so that electrons and holes can flow to the continuous AR through bipolar diffusion. Such structures can be realized using either selective area growth (SAG) or ion-implantation techniques. Our simulations show that current crowding can almost be eliminated by using the LHJ structure and that it is possible to reduce the resistive heating of the devices by further improvements using suitable material composition gradings.

### 3.2. Realization of LHJ Using Ion Implantation

The conventional approach to realize laterally doped structures e.g., in silicon industry is ion implantation. However, ion implantation doping in GaN is challenging due to several reasons. First of all, the ionization energy of the implanted materials in GaN is considerably large and thereby results in low activation efficiency [48,49]. Secondly, relatively high temperatures are typically required to achieve activation of both n- and p-type implanted dopants [50,51]. Moreover, ion implantation technique inflicts damage to the GaN lattice and damage removal is not straightforward [51]. Nevertheless, the possibility of creating a n-GaN layer on p-type GaN with reasonable carrier concentration 5 × 10${}^{19}$ cm${}^{-3}$ has been demonstrated when Si-implanted p-type GaN was annealed in N${}_{2}$ ambience [49].

**Figure 6:**
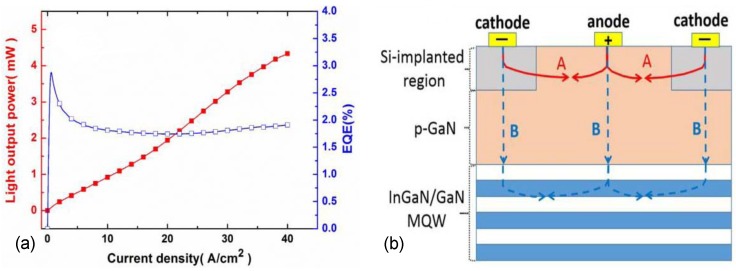
Results from a GaN based LED with a lateral pn-junction formed by selective area Si-Ion implantation [52]. The LED operates by bipolar diffusion injection. (**a**) typical current density-dependent light output power and external quantum efficiency (EQE); (**b**) schematic of the current paths in the LEDs. The separation between the contacts of the structure is approximately 50 μm. Reproduced from [52], with the permission of ©IEEE Publishing 2017.

Despite the challenges associated with ion implantation, a device structure based on a lateral GaN pn-junction was introduced very recently by Lee et al. [52]. They demonstrated a laterally doped GaN-based light-emitting diode with the InGaN/GaN QWs placed under a lateral array of GaN pn-homojunctions shown in Figure 6b. A and B lines denote the current paths of drift and diffusion current, respectively. Patterned n-doped regions were formed using selective-area Si implantation onto a MOCVD grown p-GaN cap layer followed by thermal annealing in N${}_{2}$. The resulting lateral heterojunction structure was utilized to serve as a carrier injector for the planar InGaN/GaN MQW stack placed underneath the p-GaN.

Figure 6a illustrates the current-dependent light output power and EQE of the fabricated LED at room temperature. The device operates most efficiently at low current densities (<5 A/cm${}^{2}$) and exhibits a clear efficiency droop. At intermediate injection currents (<20 A/cm${}^{2}$ corresponding to forward voltage of <15 V), the device operates at a nearly resistive regime and its efficiency decreases monotonically. At large current densities (>20 A/cm${}^{2}$), however, the efficiency starts to increase. The reasons for such behavior are not presently known. It is, however, likely that the main reason for the low maximum EQE is related with the damage caused by the ion implantation process and lack of light extraction, while the unconventional droop features may be associated with the high thermal load and large bias voltages exceeding 10 V at current densities larger than 10 A/cm${}^{2}$.

The advantage of the ion implantation doped structure is the ability to form selectively doped lateral pn-junctions without a dry etching procedure that can chemically alter the GaN surface and make the fabrication of ohmic contacts more challenging [53]. In the considered implanted structure, a heavily Si-doped n${}^{+}$-InGaN top layer was created on the p-GaN layer. After Si-ion implantation with 1 × 10${}^{16}$ cm${}^{-2}$ dosage and 70 keV energy, the implanted Si ions distributed an average depth of approximately 60 nm from the top surface layer. Annealing samples at 1000 ${}^{\circ }$C in N${}_{2}$ ambient activates the implanted Si ions in the p-GaN layer and converts p-GaN layer with a hole concentration of 3 × 10${}^{17}$ cm${}^{-3}$ into n-GaN layer with sheet electron concentration of 3 × 10${}^{14}$ cm${}^{-2}$ [52].

### 3.3. Selective Area Growth as a Method to Realize a Lateral Pn-Junction

In addition to ion implantation, selective area growth (SAG) can also be used to fabricate patterned areas of semiconductor material. The SAG of arsenide and phosphide III-V materials has been analyzed quite extensively and utilized as a major method for nanowire growth [54,55,56]. In III-N technology, SAG has been employed mostly in epitaxial lateral overgrowth (ELOG) methods that were developed to reduce threading dislocations in heteroepitaxial growth [57,58,59]. In contrast with ion implantation, the extensively characterized [60,61,62,63,64] defect-free GaN layers grown by lateral epitaxial over-growth can provide a more beneficial solution to realize LHJ structures [65].

**Figure 7:**
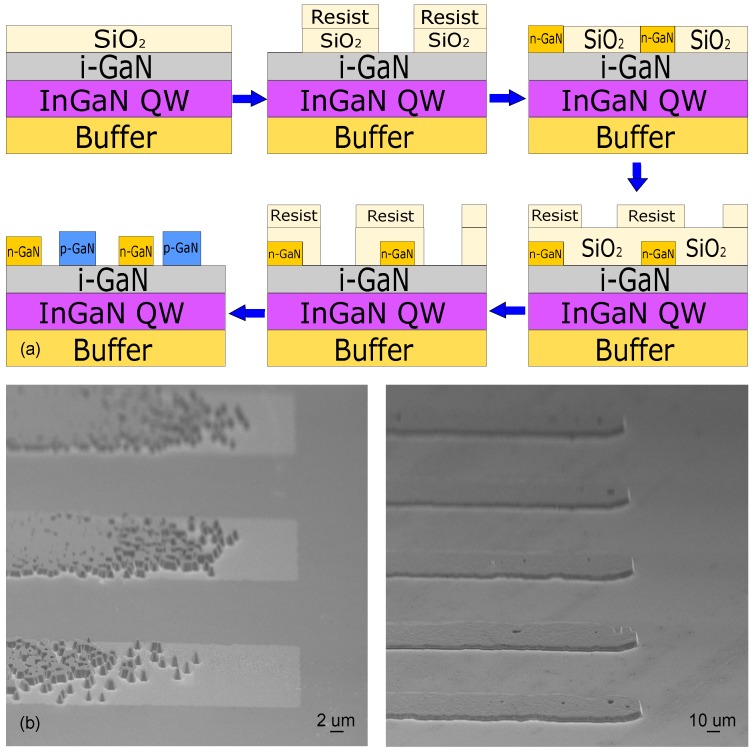
(**a**) the main steps of the LHJ LED fabrication process by selective area growth (SAG); (**b**) typical SEM images of SAG grown n-GaN fingers showing the effect of chemical patterning residues on growth (**left**) as well as the properly grown fingers (**right**).

For the SAG of p- and n-type GaN layers needed to fabricate the structure shown in Figure 5, we utilized a 6 × 2” Aixtron close-coupled showerhead MOCVD system. Trimethylgallium (TMGa), trimethylaluminum (TMAl), and trimethylindium (TMIn) were used as precursors for gallium, aluminum, and indium, respectively. Ammonia (NH${}_{3}$) was used as a precursor for the nitrogen (N${}_{2}$). Disilane (Si${}_{2}$H${}_{6}$) and bis(cyclopentadienyl)magnesium (Cp${}_{2}$Mg) were used for n- and p-type doping, respectively. The carrier concentrations of the layers at room temperature were 2 × 10${}^{17}$ cm${}^{-3}$, 5 × 10${}^{18}$ cm${}^{-3}$, and 5 × 10${}^{16}$ cm${}^{-3}$ for p-GaN, n-GaN, and i-GaN, respectively. All structures were fabricated on 2-inch c-Al${}_{2}$O${}_{3}$ wafers with a 5 μm unintentionally doped GaN buffer layer, followed by a standard 5 well InGaN/GaN MQW active region and a 120 nm i-GaN capping layer. These template structures were then used as substrates for studying the n-GaN and p-GaN SAG processes. The SAG mask was fabricated by standard lithography techniques and a SiO${}_{2}$ layer deposited by plasma-enhanced chemical vapor deposition (PECVD). The mask openings fingers and spacings in SiO${}_{2}$ growth mask were varied from 2 μm to 20 μm. The n-type SAG layer and the p-type SAG layer are then grown in separate epitaxial processes. Process flow is schematically illustrated on Figure 7a.

The epitaxial overgrowth requires a proper cleaning step of the top layer from the resist residue or SiO${}_{2}$ residue in mask openings. An ill-prepared sample can lead to not well-faceted growth, threading dislocations or non-uniform growth as shown on the left image in Figure 7b. These defects have no significant influence on luminescence from optical pumping, but, for electrically excited samples, they could dramatically increase the electrical resistance of the interfaces. An interesting feature of SAG in submillimeter scale is given by the different vertical and lateral overgrowth rates in mask openings. However, these strong geometrical effects can be controlled with pattern mask geometry [66].

**Figure 8:**
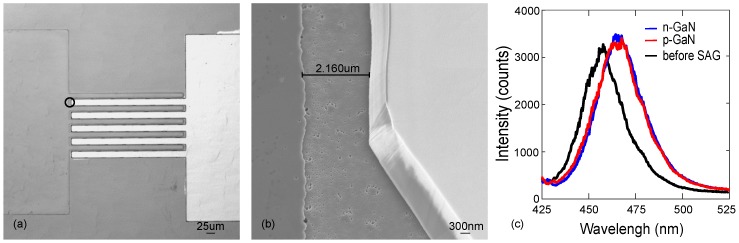
(**a**) microscope image of the LHJ structure fabricated by SAG, with separately grown n- (**feature on the left**) and p-GaN (**feature on the right**) regions; (**b**) SEM image of the area circled in (**a**) with the measured distance between the grown materials [47]; (**c**) photoluminescence spectra before and after the SAG of n-GaN and p-GaN layers on the device template, confirming that SAG does not effect the luminescence of the MQW. Figure (**c**) reproduced from [42], with the permission of ©Wiley-VCH Verlag GmbH & Co. KGaA. Publishing 2017.

In Refs. [42,47], we showed first results from structure with separately grown n- and p-GaN regions shown on Figure 8a,b. Figure 8a shows the n-type layer (mesa and fingers on the left) and an opening in the SiO${}_{2}$ mask made for the p-GaN region (mesa and fingers on the right). Figure 8b shows an SEM image from the circled area in Figure 8a for a structure, where the SAG of both the n- and p-type GaN regions has been completed and the SiO${}_{2}$ mask has been removed. Figure 8c further shows the PL from the samples excited using a pump laser at 405 nm before and after SAG of p- and n-type GaN. Based on the PL measurements, SAG does not notably affect the luminescence of the MQW, suggesting that SAG provides a promising method to fabricate laterally doped GaN devices.

Simulations presented in the papers [42,47] compare the lateral current spreading and current crowding properties of high power GaN LEDs based on conventional DHJ structures and structures based on the DDCT principle. As a result, we showed that using a single-side graded active region both facilitates the current transport in the LHJ device and leads to only a modest efficiency droop by increasing the effective thickness of the active region. Moreover, comparing the operation of the LHJ structure with conventional LEDs and an ideal vertical LED showed that the LHJ structure shows practically no added differential resistance or efficiency loss due to lateral current crowding.

## 4. Outlook of DDCT-Based LEDs

**Figure 9:**
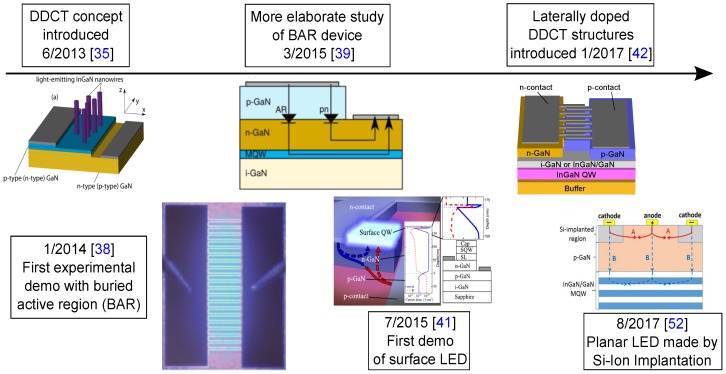
Timeline chart of historical evolution diffusion-driven charge transport concept. Reproduced from [35], with the permission of ©AIP Publishing 2013. Reproduced from [38], with the permission of ©AIP Publishing 2014. Reproduced from [39], with the permission of ©IEEE Publishing 2015. Reproduced from [41], with the permission of ©AIP Publishing 2015. Reproduced from [52], with the permission of ©IEEE Publishing 2017.

The fundamental difference between the DDCT-structure and the conventional DHJ-structure is that in the first one the AR is not sandwiched inside a pn-junction. Instead, the structure is designed so that the AR is totally separate from the pn-junction and located within the diffusion length of carriers (at most a few to a few tens of microns in absence of potential barriers) from the junction. The spatial and functional separation of the pn-junction and the AR enable a fundamentally different starting point for device design, and therefore enables very different solutions to carrier injection to a semiconductor surface than what is available using DHJ. The DDCT-scheme can be thought to provide a new and general method to transport carriers to or from the surface and adjacent materials. The main requirement for the DDCT method is that the AR can act as a carrier drain or source, which then induces the diffusion current. Therefore, the band-gap of the surface emitter generally needs to be smaller and the carrier life time shorter than in the pn-diode material. In this review article, we concentrated on DDCT of GaN LEDs (Figure 9). However, the DDCT model is in principle equally applicable for any other materials and light-emitting/absorbing devices. A particularly interesting possibility is to fabricate the pn-junction from an indirect band gap material (such as Si), which then excites a light emitting AR on the device surface.

Using the DDCT-scheme, emitters based on e.g., near surface QWs, surface NWs, QDs and ML emitting materials can be fabricated without top contacts as shown in Figure 1. III-N-based NWs grown on low cost, large-area substrates hold promises in applications in solid state lighting and full-color displays [67], and InAs and InP NWs grown on Si as near infra-red emitters [68]. Emission wavelengths ranging from UV to blue have been demonstrated using GaN-based heterostructures with EQE 80% [69], while the expansion of the LED emission wavelength to the green and then to the yellow spectral range leads to much lower maximum EQE values, namely 40–50% [70] and 20% [71], respectively. The efficiency dip in the green region is known as the “green gap” [64,72,73]. The small diameter InN-NWs are considered as one candidate technology [74] that, combined with the DDCT concept, can bridge the green gap. Additionally, room temperature phosphor-free white-light emission in the mW range has been realized by GaN nanowire LEDs [75].

Clear strengths and opportunities provided by the DDCT concept are (1) utilizing NWs, QDs and 2D-materials as the top active region to enhance LED performance and enable novel devices for optoelectronic applications; (2) eliminate current crowding and resistive losses that limit LEDs in high-power applications. However, realization of LHJ-based structures requires development of new fabrication techniques and more detail understanding of potential barriers in semiconductor heterostructures. Especially in LHJ devices, crystal damage during Si-Ion implantation and defects formed during selective area growth present a significant challenge for low resistivity electrical path through interfaces.

More detailed exploration of the possibilities to use DDCT in realizing several new types of devices calls for extensive experiments as well as developing advanced device simulation models beyond state-of-the-art. For example, as DDCT enables integrating new materials such as colloidal QDs and 2D materials on semiconductor surfaces, controlled experiments and physical simulation models need to be designed to study carrier transport across the interfaces between the semiconductor and the surface structures. On the other hand, the standard semiconductor device simulations we have mainly relied on this far have shown remarkable qualitative agreement with experiments on DDCT in the absence of active surfaces. This suggests that the DDCT concept may provide substantial added value for developing several next generation optoelectronic devices, in agreement with the presently available simulations.

## 5. Conclusions

Breaking free of the strict and long-lived limitation to sandwich the active region within a pn-junction provides new possibilities for the design and development of emerging next generation optoelectronic devices. This paper introduced the brief history of the diffusion driven charge transport concept, which allows the spatial separation of the pn-junction and the active region. Therefore, DDCT holds the promise to electrically excite new light emitting materials on device surfaces, for example. While the fundamental possibility of DDCT is easily visible from the diffusion terms of the basic semiconductor transport equations, its technological relevance is much more difficult to assess.

More specifically, in this article, we reviewed several technologically relevant demonstrations of the concept in various light emitting structures. We began by introducing the theoretical background of the DDCT concept applied to a NW LED. Simulations clearly indicated that DDCT can be used to inject carriers into an AR outside of a pn-junction and encouraged experimental studies of the topic. The structure with an MQW buried under the pn-junction provided the first experimental proof of the DDCT concept. For even stronger evidence, the S-LED structure with a QW located on the device surface was fabricated and characterized. Bipolar diffusion was the only mechanism that could explain the blue luminescence from the QW, and therefore these works fully established DDCT as a new way to realize light emitting devices. The remaining part of this review discussed the ongoing work around new potentially more efficient DDCT-based devices with lateral heterojunctions. Si-Ion implantation and selective area growth were presented as the most promising ways to form the lateral pn-junctions. We also briefly discussed the outlook of using DDCT in developing new types of nanowire LEDs and other emerging possibilities enabled by DDCT.

In order to gain further technological traction, however, the next development steps of the concept will involve both studying the possibility to optimize the presently introduced structures as well as to demonstrate entirely new approaches to realize e.g., applications involving NWs or nanoplasmonics. If the predictions of the simulations carried out on the DDCT-structures this far turn out to be reliable, the deployment of the DDCT concept could lead to dramatic improvements in the ability to harness the emerging nanomaterials for practical applications.

## Abbreviations

The following abbreviations are used in this manuscript:

LEDs	Light-Emitting Diodes
DHJ	Double Heterojunction
DDCT	Diffusion-Driven Charge Transport
AR	Active Region
LHJ	Lateral Heterojunction
GaN	Gallium Nitride
QW	Quantum Well
MQW	Multi-Quantum Well
NW	Nanowires
QDs	Quantum-Dots
InGaN	Indium Gallium Nitride
DILED	Diffusion Injected Light Emitting Diode
S-LED	Surface Light Emitting Diode
SAG	Selective Area Growth
MOCVD	Metal-Organic Chemical Vapor Deposition
PECVD	Plasma-Enhanced Chemical Vapor Deposition
EQE	External Quantum Efficiency
